# A Culturally and Linguistically Tailored Intervention to Improve Diabetes-Related Outcomes in Chinese Americans With Type 2 Diabetes: Pilot Randomized Controlled Trial

**DOI:** 10.2196/78036

**Published:** 2025-10-27

**Authors:** Jing Liu, Jiepin Cao, Yun Shi, Mary Ann Sevick, Nadia Islam, Naumi Feldman, Huilin Li, Chan Wang, Yanan Zhao, Kosuke Tamura, Natalie Levy, Nan Jiang, Ziqiang Zhu, Yulin Wang, Jia Hong, Lu Hu

**Affiliations:** 1 Department of Population Health NYU Langone Health New York City, NY United States; 2 Charles B. Wang Community Health Center New York City, NY United States; 3 Division of Intramural Research National Institute on Minority Health and Health Disparities National Institutes of Health Bethesda, MD United States; 4 Department of Medicine NYU Langone Health New York City, NY United States; 5 Wellsure Medical Practice New York City, NY United States; 6 Yulin Medicine PLLC New York City, NY United States; 7 Hong Jia Medical PC New York City, NY United States

**Keywords:** type 2 diabetes, diabetes self-management, health equity, minority health, immigrant health, mobile health, social media, community health worker, social needs, social determinants of health

## Abstract

**Background:**

Chinese Americans with type 2 diabetes (T2D) face many linguistic, cultural, and social determinants of health–related barriers to accessing evidence-based diabetes interventions. Our team developed the culturally and linguistically tailored Chinese American Research and Education (CARE) program to provide evidence-based diabetes education and support to this group and demonstrated the feasibility, acceptability, and potential efficacy of the intervention on improving hemoglobin A_1c_ levels. However, it remains unclear whether the CARE program also improves diabetes self-efficacy and psychosocial outcomes in the same study sample.

**Objective:**

This is a secondary analysis to examine the potential efficacy of the CARE program on secondary outcomes, including diabetes self-efficacy, self-care activities, beliefs in diabetes self-care activities, and diabetes distress among Chinese Americans with T2D.

**Methods:**

A 2-arm, pilot randomized controlled trial was conducted to evaluate the CARE program between March 1, 2021, and April 21, 2023. The trial included 60 Chinese Americans aged 18 to 70 years who had a diagnosis of T2D and a baseline hemoglobin A_1c_ level of 7% or higher. Participants were recruited from various health care settings in New York City, including community health centers, private primary care providers, and NYU Langone Health and its affiliates, and were randomly assigned to either the CARE intervention group (n=30) or a waitlist control group (n=30). The intervention consisted of 2 culturally and linguistically tailored educational videos per week for 12 weeks, covering diabetes self-care topics such as healthy eating, physical activity, and medication adherence. These videos were delivered via the WeChat app. In addition, community health workers provided support calls to assist them in setting goals, problem-solving, and addressing social determinants of health barriers every 2 weeks. Secondary outcomes included patient self-reported diabetes self-efficacy, self-care activities, beliefs in diabetes self-care activities, and diabetes distress. Outcomes were assessed at baseline, 3 months, and 6 months.

**Results:**

Participants had a mean age of 54.3 (SD 11.5) years and 62% (37/60) were male, 78% (47/60) were married, 58% (35/60) were employed, 70% (42/60) had a high school education or lower, and 88% (53/60) reported limited English proficiency. Intervention participants demonstrated statistically significant improvements in self-efficacy at 3 months (estimated difference in change: 8.47; 95% CI 2.44-14.5; adjusted *P*=.02), diabetes distress at 6 months (estimated difference in change: –0.43; 95% CI –0.71 to –0.15; adjusted *P*=.009), and adherence to a healthy diet at both 3 months (estimated difference in change: 1.61; 95% CI 0.46-2.75; adjusted *P*=.02) and 6 months (estimated difference in change: 1.64; 95% CI 0.48-2.81; adjusted *P*=.02).

**Conclusions:**

The culturally and linguistically tailored intervention showed promise in improving self-efficacy and diabetes self-care activities among Chinese Americans with T2D, warranting validation through a large-scale randomized controlled trial.

**Trial Registration:**

ClinicalTrials.gov NCT03557697; https://clinicaltrials.gov/study/NCT03557697.

## Introduction

Chinese Americans are among the fastest-growing racial and ethnic minority groups in the United States and face a significant burden of type 2 diabetes (T2D). The prevalence of T2D in this population is remarkably higher than in that of non-Hispanic White Americans [1,2] despite generally lower BMI levels [3,4]. Beyond the high prevalence, Chinese Americans often experience worse outcomes and complications due to social determinants of health (SDOH) barriers, such as limited access to health care services, financial constraints, limited English proficiency, and cultural barriers.All these factors can impede adherence to recommended T2D self-care practices to achieve optimal glycemic control.

Self-care behaviors are essential components of effective T2D management, such as medication adherence, healthy eating, regular physical activity, and routine blood glucose monitoring [5,6]. Self-efficacy, referred to as an individual’s confidence in their ability to manage the illness [6,7], is a well-established predictor of engagement in these self-care practices and diabetes distress [8,9]. Higher levels of self-care behaviors and self-efficacy have been associated with improved glycemic control [5,9]. Notably, a prior study of 3067 older participants from the 2009 California Health Interview Survey demonstrated that Asian individuals with diabetes exhibit the lowest levels of diabetes self-efficacy when compared to non-Hispanic White, American Indian or Alaska Native, African American, and Hispanic or Latino populations [10]. Asian individuals were also significantly less likely than White individuals to regularly test their blood glucose or receive foot examinations in accordance with clinical guidelines [11,12]. For Chinese Americans, the largest Asian subgroup, enhancing self-efficacy and self-care practices may be particularly impactful, as this population often faces significant cultural and linguistic barriers to accessing diabetes services. These disparities underscore the pressing need for easily accessible, tailored interventions to strengthen self-efficacy and promote sustained self-care behaviors among Chinese Americans.

Diabetes self-management education and support (DSMES) programs have been shown to be effective in promoting diabetes self-efficacy and self-care behaviors and improving glycemic control among patients with T2D [13,14]. Yet, these evidence-based programs are underused by Chinese Americans. Existing DSMES programs often lack cultural relevance, making it difficult for Chinese Americans to apply the recommendations to their diet [15,16]. Limited English proficiency also poses a substantial communication barrier between Chinese American patients and health care providers [17]. Furthermore, the requirement for in-person visits in traditional DSMES programs can be impractical for many Chinese Americans, who often face transportation challenges and work long hours [18,19]. Therefore, supportive interventions that are easily accessible and address the cultural and linguistic needs of this population are required.

To address these challenges, we developed the Chinese American Research and Education (CARE) program by culturally and linguistically adapting the DSMES intervention specifically for Chinese Americans with T2D [20]. The CARE adaptation was guided by both the Cultural Adaptation Model [21] and the Ecological Validity Model [22]. The CARE program is a 12-week intervention grounded in Social Cognitive Theory, which posits that self-efficacy is a crucial determinant of behavior change [23]. It includes 24 brief videos covering key topics in T2D self-management, such as diabetes basics, healthy eating, physical activity, medication adherence, glucose monitoring, and behavioral techniques (eg, goal-setting and problem-solving). Examples of the CARE videos are presented in Multimedia Appendix 1. Additionally, community health workers provide supportive phone calls every 2 weeks to participants to assist them in setting goals and problem-solving and address specific SDOH challenges encountered in their self-management efforts.

The CARE program was previously evaluated through a pilot randomized controlled trial (RCT) to assess its feasibility, acceptability, and potential efficacy on primary outcomes, including hemoglobin A_1c_ (HbA_1c_) levels, BMI, physical activities, and diet, as reported elsewhere [24]. The present study is a secondary analysis of the CARE trial, focusing on its potential impact on diabetes self-efficacy, self-care activities, beliefs in diabetes self-care activities, and diabetes distress among Chinese immigrants with T2D. We hypothesize that participants in the CARE intervention group will demonstrate significantly greater improvements in diabetes self-efficacy, self-care activities, and beliefs in diabetes self-care activities and reductions in diabetes distress compared to those in the waitlist control group at both the 3- and 6-month follow-ups.

## Methods

### Design

The current study is a secondary analysis of the pilot RCT that examined the potential efficacy of the CARE program on HbA_1c_ levels. A total of 60 Chinese American participants with T2D were enrolled and randomly assigned in a 1:1 ratio to either the CARE intervention group or a waitlist control group. This study follows the CONSORT (Consolidated Standards of Reporting Trials) guidelines (Multimedia Appendix 2) [25]. Detailed descriptions of the pilot RCT have been published elsewhere [20,24].

### Participants and Recruitment

Participants were recruited from March 2021 to March 2022 from various New York City–based health care facilities, including the Charles B Wang Community Health Center, private primary care providers, and NYU Langone Health and its affiliates. Recruitment strategies included (1) distributing study flyers, (2) direct referrals from health care providers, and (3) NYU Langone Health electronic medical records. Study staff members screened interested individuals by telephone to confirm eligibility.

Participants were eligible if they met the following criteria: (1) self-identified as Chinese immigrants or Chinese Americans; (2) were between the ages of 18 and 70 years; (3) were proficient in Mandarin, one of the most widely spoken Chinese languages; (4) had a diagnosis of T2D with a most recent HbA_1c_ level of 7% or higher at baseline; (5) used WeChat, a free social media app widely used among Chinese Americans; (6) owned a smartphone or tablet or agreed to use a study-provided device; and (7) were interested in receiving T2D-related videos via WeChat.

Exclusion criteria included significant uncorrected vision or hearing impairments, reluctance to comply with random assignments, pregnancy or plans to become pregnant during the study, breastfeeding, and residing in environments impeding T2D self-management, such as nursing homes.

### Intervention

#### CARE Intervention Group

Based on Social Cognitive Theory [23], the CARE intervention aims to enhance self-efficacy in T2D self-management by addressing mastery experiences, social modeling, verbal persuasion, and physiological states. Participants in the intervention group received 2 culturally and linguistically tailored DSMES videos per week for 12 weeks (3 months) through WeChat. These videos covered various T2D self-care topics, including diabetes basics, healthy eating, physical activity, medication adherence, glucose monitoring, and behavioral techniques such as goal-setting and problem-solving. These videos were delivered in Mandarin. Additionally, participants received support calls from trained community health workers every 2 weeks, who assisted them in setting goals, problem-solving, and addressing SDOH barriers. After 12 weeks of video education and support calls, participants in the intervention group received usual care (3-6 months).

#### Waitlist Control Group

Waitlist control group participants received usual care. After the study ended (at 6 months), they were given access to the same DSMES educational videos via WeChat as an incentive.

### Blinding

The blinding of participants and community health workers who delivered the intervention was not feasible due to the nature of the intervention. However, the data management and statistical analyses were conducted by analysts who were blinded to the allocation.

### Measures

#### Demographic Information

Information on age, gender, marital status, education, annual income, employment status, English proficiency, and years of residency in the United States were collected at baseline.

#### Self-Efficacy for Diabetes

The Stanford 8-item Self-Efficacy for Diabetes scale was used to evaluate participants’ confidence in managing their diabetes through various self-care behaviors, including diet, physical activity, blood glucose monitoring, and self-control [26]. Participants rated their confidence on a 10-point Likert scale, ranging from 1 (not at all confident) to 10 (totally confident). The total score was calculated, with higher scores indicating greater self-efficacy.

#### Diabetes Self-Care Activities

The Summary of Diabetes Self-Care Activities was used to assess specific diabetes self-care activities across general diet (ie, following a healthful eating plan), specific diet (ie, intake of fruits and vegetables and high-fat foods), exercise, self-monitoring of blood glucose, foot care, and medication adherence [27]. Participants indicated how many days in the past week they engaged in these specified behaviors. The mean number of days adherent to each type of diabetes self-care activity per week was calculated.

#### Beliefs in Diabetes Self-Care Activities

Participants rated the importance of 5 self-care behaviors related to diabetes-following a diabetic diet, exercising, self-monitoring blood glucose, taking medication, and checking their feet. Each behavior was rated on a 5-point Likert scale, where 1 indicated “not important” and 5 indicated “very important” [28].

#### Diabetes Distress

The Diabetes Distress Scale was used to evaluate 4 domains of diabetes-related distress: emotional burden, physician-related distress, regimen-related distress, and interpersonal distress [29]. This 17-item scale required participants to rate the extent to which each item was problematic for them in the past month using a 6-point Likert scale ranging from 1 (no problem) to 6 (serious problem). Mean item scores were calculated, with higher scores indicating a greater level of distress.

### Sample Size Calculation

This study is a pilot RCT primarily aimed at establishing the feasibility, acceptability, and potential efficacy of the CARE program on HbA_1c_ levels. The sample size of this pilot RCT was determined based on HbA_1c_ levels, which is the most commonly evaluated primary outcome when testing DSMES programs. According to Whitehead et al [30], in order to detect a small group difference of 0.2% in HbA_1c_ levels with 80% power and 5% type I error in a future full-scale RCT, we would need to recruit 50 participants in a pilot RCT. To account for a 20-25% attrition rate observed in previous studies with Chinese Americans [31-34], we aimed to enroll 60 participants, with 30 in each of the intervention and control groups.

### Data Analysis

We followed the intention-to-treat principle in data analysis. We used chi-square tests and independent 2-sample *t* tests to compare variables between the intervention and control groups. We applied piecewise linear mixed models to compare changes in outcomes over 0-6 months between the intervention and control groups. In these models, the 2 time periods (0-3 months and 3-6 months), the intervention group, and the intervention × time period interactions were modeled as fixed effects. We considered the participant as a random effect and included variables that showed statistically significant differences as covariates for adjustment. We applied the Tukey method to adjust *P* values for differences across time points within each group and the Bonferroni method to adjust *P* values for differences in changes over time between the 2 groups. We used R software (version 4.4.2; R Foundation for Statistical Computing) for statistical analyses and set the significance level at .05 for 2-tailed tests.

In addition to statistical significance, this study used the minimal clinically important difference (MCID) to assess the clinical significance of between-group differences. In this study, an MCID of 0.25 was considered clinically significant for the Diabetes Distress Scale [35]. As no established MCIDs are available for the Stanford 8-item Self-Efficacy for Diabetes Scale, the Summary of Diabetes Self-Care Activities, or the Beliefs in Diabetes Self-Care Activities, a threshold of 6% to 10% of the total score was used for these scales, as recommended by previous research [36]. Therefore, an MCID of 4.8-8.0 points on the Self-Efficacy for Diabetes Scale (total score: 80), 0.42-0.7 on the Summary of Diabetes Self-Care Activities (total score: 7), and 0.3-0.5 on the Beliefs in Diabetes Self-Care Activities (total score: 5) were used to evaluate the clinical significance of these outcomes. Clinical significance was determined by comparing the 95% CI of the observed differences to the MCID threshold for each scale: differences were considered “clinically significant” if the lower bound of the 95% CI exceeded the threshold, “may be significant” if the 95% CI included the threshold, and “not clinically significant” if the upper bound of the 95% CI was below the threshold [37].

### Ethical Considerations

This study was conducted in accordance with the principles of the Declaration of Helsinki. The study was reviewed and approved by the NYU Grossman School of Medicine Institutional Review Board (IRB# s18-00609). All participants took part in the study voluntarily. Informed consent was obtained from all participants prior to their enrollment in the study. Participants’ identifiable information was deidentified before data analysis to ensure their privacy and confidentiality. Participants received US $90 for completing the baseline, 3-month, and 6-month follow-ups. Participants from both groups were also compensated US $5 per week (up to US $60) to offset mobile data costs related to viewing intervention videos. The compensation was provided via a ClinCard.

## Results

### Sample Characteristics

**Figure 1:**
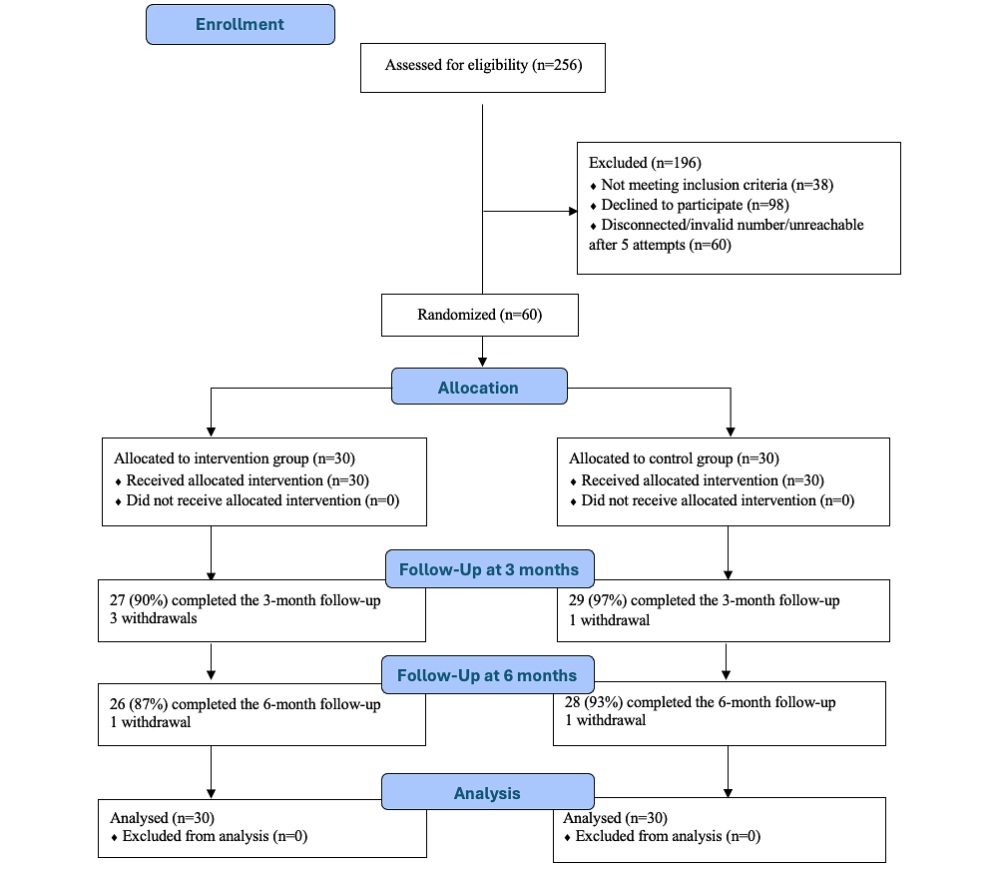
The CONSORT flow diagram.

A CONSORT flow diagram is presented in Figure 1. The mean age of participants was 54.3 (SD 11.5) years, with 62% (37/60) self-identifying as male, 78% (47/60) being married, 58% (35/60) being currently employed, 70% (42/60) having a high school education or lower, 75% (45/60) reporting an annual household income under $55,000, and 88% (53/60) indicating limited English proficiency. At baseline, the mean HbA_1c_ level was 8.2% (SD 1.2%) and the mean BMI was 26.5 (SD 5.1) kg/m² (Asian individuals with a BMI of 23 kg/m² or greater are considered to be overweight). There were no significant differences in these demographic characteristics between the intervention and control groups. See Table 1 for details.

**Table 1:** Sample characteristics (N=60).

Characteristics			Total (N=60)		CARE^a^ group (n=30)		Control group (n=30)		*P* value	
Age (years), mean (SD)			54.3 (11.5)		55.6 (11.5)		52.9 (11.4)		.36	
**Gender, n (%)**										>.99
	Men	37 (62)		18 (60)		19 (63)				
	Women	23 (38)		12 (40)		11 (37)				
Being married, n (%)			47 (78)		23 (77)		24 (80)		>.99	
**Education, n (%)**										.40
	≤ high school education	42 (70)		19 (63)		23 (77)				
	> high school education	18 (30)		11 (37)		7 (23)				
**Annual household income (US $),** **n (%)**										.80
	≤55,000	45 (75)		23 (77)		22 (73)				
	>55,000	12 (20)		5 (17)		7 (23)				
	Missing	3 (5)		2 (7)		1 (3)				
**Employment status, n (%)**										.30
	Employed	35 (58)		14 (47)		21 (70)				
	Unemployed	10 (17)		7 (23)		3 (10)				
	Retired	13 (22)		8 (27)		5 (17)				
	Other	2 (3)		1 (3)		1 (3)				
**English proficiency, n (%)**										.64
	Very well	6 (10)		2 (7)		4 (13)				
	Limited English proficiency	53 (88)		28 (93)		25 (83)				
	Missing	1 (2)		0 (0)		1 (3)				
Duration of residency (years), mean (SD)			19.2 (10.8)		20.4 (11.4)		17.9 (10.2)		.38	
Missing duration of residency data, n (%)			2 (3)		1(3)		1(3)		—^b^	
HbA_1c_ (%), mean (SD)			8.24 (1.24)		8.35 (1.30)		8.12 (1.19)		.46	
Body weight (lbs), mean (SD)			160 (42.9)		154 (34.0)		166 (50.3)		.31	
BMI (kg/m²), mean (SD)			26.5 (5.1)		26.0 (4.3)		27.1 (5.8)		.45	
Self-efficacy for diabetes, mean (SD)			55.1 (14.3)		53.3 (14.2)		56.9 (14.5)		.35	
Missing self-efficacy for diabetes data, n (%)			2 (3)		1 (3)		1 (3)		—	
**Self-care activities**, mean (SD)										
	General diet	3.91 (2.59)		3.60 (2.67)		4.22 (2.51)		.36		
	Specific diet	2.69 (1.72)		2.62 (1.84)		2.77 (1.63)		.74		
	Exercise	2.88 (2.46)		2.53 (2.45)		3.23 (2.45)		.27		
	Blood glucose testing	2.52 (2.72)		2.70 (2.87)		2.33 (2.59)		.66		
	Missing blood glucose testing data, n (%)	16 (27)		7 (23)		9 (3)		—		
	Foot Care	1.30 (1.81)		1.64 (2.00)		0.967 (1.56)		.16		
	Missing foot care data, n (%)	1 (2)		1 (3)		0		—		
	Medication	6.70 (0.91)		6.79 (0.82)		6.61 (0.99)		.44		
	Missing medication data, n (%)	2 (3)		1 (3)		1 (3)		—		
	Beliefs in diabetes self-care activities, mean (SD)	4.56 (0.56)		4.57 (0.58)		4.54 (0.55)		.85		
	Missing beliefs in diabetes self-care activities data, n (%)	2 (3)		1 (3)		1 (3)		—		
	Diabetes distress, mean (SD)	1.74 (0.84)		2.09 (1.01)		1.40 (0.43)		.002		
	Missing diabetes distress data, n (%)	1 (2)		1 (3)		0		—		

^a^CARE: Chinese American Research and Education.

^b^Not applicable.

### Self-Efficacy for Diabetes

Table 2 presents the changes in participants’ outcomes at baseline, 3 months, and 6 months. At baseline, the intervention group had a self-efficacy score of 53.3 (SD 14.2), while the control group scored 56.9 (SD 14.5). This difference was not statistically significant (*P*=.35).

Compared to the control group, participants in the intervention group demonstrated a statistically significantly greater increase in self-efficacy for diabetes management at 3 months (estimated difference in change: 8.47; 95% CI 2.44 to 14.5; adjusted *P*=.02). The observed between-group difference in self-efficacy at the 3-month follow-up may indicate a clinically meaningful improvement. However, at 6 months, the increase was not statistically or clinically significant (estimated difference in change: 5.87; 95% CI –0.23 to 11.98; adjusted *P*=.18).

**Table 2:** Outcomes of interests at baseline, 3 months, and 6 months.

Outcomes by time period (months)		CARE^a^				Control					Difference of change (CARE – control)			
		Est^b^	95% CI	Raw *P* value^c^	Adj *P* value^d^	Est	95% CI	Raw *P* value^c^	Adj *P* value^d^	Est		95% CI	Raw *P* value^c^	Adj *P* value^e^
**Self-efficacy for diabetes**														
	0-3	5.76	(1.45 to 10.06)	.009	.03	–2.72	(–6.93 to 1.5)	.21	.41	8.47		(2.44 14.5)	.006	.02
	3-6	0.32	(–4.07 to 4.72)	.88	.99	2.92	(–1.31 to 7.16)	.17	.36	–2.6		(–8.7 to 3.5)	.40	>.99
	0-6	6.08	(1.72 to 10.44)	.007	.02	0.21	(–4.07 to 4.48)	.92	.99	5.87		(–0.23 to 11.98)	.06	.18
**Self-care activities: general diet**														
	0-3	2.04	(1.21 to 2.86)	< .001	< .001	0.43	(–0.37 to 1.23)	.29	.54	1.61		(0.46 to 2.75)	.007	.02
	3-6	–0.3	(–1.15 to 0.54)	.48	.76	–0.34	(–1.15 to 0.48)	.41	.70	0.04		(–1.14 to 1.21)	.95	>.99
	0-6	1.74	(0.9 to 2.57)	< .001	< .001	0.09	(–0.72 to 0.91)	.82	.97	1.64		(0.48 to 2.81)	.006	.02
**Self-care activities: specific diet**														
	0-3	0.17	(–0.47 to 0.81)	.60	.86	–0.31	(–0.93 to 0.32)	.33	.59	0.48		(–0.42 to 1.38)	.29	.88
	3-6	0.42	(–0.24 to 1.08)	.21	.42	0.51	(–0.12 to 1.15)	.11	.25	–0.09		(–1.01 to 0.82)	.84	>.99
	0-6	0.59	(–0.06 to 1.24)	.07	.17	0.2	(–0.43 to 0.84)	.53	.80	0.39		(–0.52 to 1.29)	.40	>.99
**Self-care activities: exercise**														
	0-3	1.26	(0.43 to 2.1)	.003	.009	0.2	(–0.61 to 1.01)	.63	.88	1.07		(–0.1 to 2.23)	.07	.22
	3-6	0.29	(–0.56 to 1.15)	.50	.76	0.69	(–0.13 to 1.52)	.10	.22	–0.4		(–1.59 to 0.79)	.51	>.99
	0-6	1.56	(0.71 to 2.4)	< .001	.001	0.89	(0.07 to 1.72)	.03	.08	0.67		(–0.51 to 1.84)	.27	.80
**Self-care activities: blood sugar testing**														
	0-3	0.47	(–0.52 to 1.46)	.35	.61	–0.7	(–1.68 to 0.28)	.16	.33	1.17		(–0.22 to 2.56)	.09	.30
	3-6	–0.36	(–1.39 to 0.66)	.48	.76	0.56	(–0.45 to 1.58)	.27	.51	–0.93		(–2.37 to 0.51)	.20	.61
	0-6	0.1	(–0.9 to 1.11)	.84	.98	–0.14	(–1.13 to 0.86)	.79	.96	0.24		(–1.18 to 1.66)	.74	>.99
**Self-care activities: foot care**														
	0-3	–0.22	(–0.88 to 0.44)	.51	.78	0	(–0.63 to 0.63)	.99	>.99	–0.22		(–1.13 to 0.69)	.64	>.99
	3-6	0.29	(–0.38 to 0.95)	.40	.67	–0.17	(–0.81 to 0.47)	.61	.86	0.45		(–0.47 to 1.38)	.33	>.99
	0-6	0.07	(–0.6 to 0.73)	.85	.98	–0.17	(–0.81 to 0.47)	.60	.86	0.24		(–0.69 to 1.16)	.61	>.99
**Self-care activities: medication**														
	0-3	–0.03	(–0.59 to 0.53)	.92	.99	0.06	(–0.49 to 0.62)	.82	.97	–0.09		(–0.88 to 0.7)	.82	>.99
	3-6	–0.44	(–1.01 to 0.14)	.14	.30	–0.12	(–0.69 to 0.45)	.67	.91	–0.31		(–1.12 to 0.5)	.45	>.99
	0-6	–0.46	(–1.03 to 0.11)	.11	.24	–0.06	(–0.63 to 0.51)	.84	.98	–0.41		(–1.21 to 0.4)	.32	.96
**Beliefs in diabetes self-care activities**														
	0-3	0.05	(–0.15 to 0.24)	.64	.88	0.21	(0.02 to 0.4)	.03	.08	–0.17		(–0.44 to 0.11)	.24	.70
	3-6	0.1	(–0.1 to 0.3)	.35	.61	0	(–0.2 to 0.19)	.98	>.99	0.1		(–0.18 to 0.38)	.49	>.99
	0-6	0.14	(–0.06 to 0.34)	.16	.33	0.21	(0.02 to 0.4)	.04	.09	–0.07		(–0.35 to 0.21)	.63	>.99
**Diabetes distress**														
	0-3	–0.24	(–0.44 to –0.04)	.02	.047	0.01	(–0.18 to 0.2)	.93	.99	–0.25		(–0.52 to 0.03)	.08	.23
	3-6	–0.13	(–0.34 to 0.07)	.19	.39	0.05	(–0.15 to 0.25)	.62	.87	–0.18		(–0.47 to 0.1)	.2	.60
	0-6	–0.37	(–0.58 to –0.17)	< .001	< .001	0.06	(–0.13 to 0.25)	.55	.82	–0.43		(–0.71 to –0.15)	.003	.009

^a^CARE: Chinese American Research and Education.

^b^Est: estimated difference in change.

^c^Initial *P* values without adjustment.

^d^Adjusted *P* values using the Tukey method.

^e^Adjusted *P* values using the Bonferroni method.

### Self-Care Activities

At baseline, participants in the intervention group and control group did not differ in self-care activities (all *P*>.05). Specifically, participants in the intervention group followed their healthful eating plan for an average of 3.60 (SD 2.67) days per week, compared to 4.22 (SD 2.51) days per week in the control group, with no statistically significant difference (*P*=.36). For the specific diet, the intervention group followed it for 2.62 (SD 1.84) days per week, while the control group followed it for 2.77 (SD 1.63) days per week, which was not statistically significant (*P*=.74). Regarding exercise, the intervention group exercised for 2.53 (SD 2.45) days per week, compared to 3.23 (SD 2.45) days per week in the control group, with no significant difference (*P*=.27). Foot care was performed by the intervention group for 1.64 (SD 2.00) days per week, compared to 0.967 (SD 1.56) days per week in the control group, with no significant difference (*P*=.16). Finally, both groups adhered to their medication regimens similarly, with the intervention group adhering for 6.79 (SD 0.82) days per week and the control group for 6.61 (SD 0.99) days per week, with no statistically significant difference (*P*=.44).

Participants in the intervention group were more adherent to a healthy diet at 3 months (estimated difference in change: 1.61; 95% CI 0.46 to 2.75; adjusted *P*=.02) and 6 months (estimated difference in change: 1.64; 95% CI 0.48 to 2.81; adjusted *P*=.02) compared to the control group. The observed between-group differences in healthy diet at both the 3- and 6- month follow-ups may indicate a clinically meaningful improvement. There was no statistical or clinical significance in the between-group differences in other self-care activities at 3 months or 6 months, including exercise, blood glucose testing, foot care, and medication adherence (all *P*>.05).

### Beliefs in Diabetes Self-Care Activities

At baseline, both the intervention group (mean 4.57, SD 0.58) and the control group (mean 4.54, SD 0.55) recognized the importance of self-management activities for their diabetes, including following a healthy diet, exercising, self-monitoring blood glucose, taking medication, and checking their feet. The difference was not statistically significant (*P*=.85). There were no statistically or clinically significant differences in beliefs about self-management at 3 months (adjusted *P*=.70) or 6 months (adjusted *P>*.99).

### Diabetes Distress

At baseline, both the intervention group (mean 2.09, SD 1.01) and the control group (mean 1.40, SD 0.43) reported minimal levels of diabetes distress. However, participants in the intervention group reported significantly higher diabetes distress compared to those in the control group (*P*=.002).

Compared to the control group, participants in the intervention group reported a significant improvement in diabetes distress at 6 months (estimated difference in change: –0.43; 95% CI: –0.71 to –0.15; adjusted *P*=.009). The observed between-group differences in diabetes distress at the 6-month follow-up may indicate a clinically meaningful improvement. No statistically or clinically significant differences in diabetes-related distress between the intervention and control groups were observed at 3 months (adjusted *P*=.23).

## Discussion

### Principal Findings

This study found that, compared to usual care, our 12-week CARE intervention, which included weekly diabetes education videos and biweekly support calls from community health workers, demonstrated statistically significant improvements in self-efficacy at 3 months, adherence to a healthy diet at both 3 and 6 months, and diabetes distress at 6 months. These between-group improvements may be clinically significant. These findings highlight the potential benefits of the CARE program, a culturally and linguistically tailored, social media–based intervention, in improving self-efficacy and diabetes self-care activities among Chinese Americans with T2D. The results suggest that culturally and linguistically tailored interventions may be a promising strategy for addressing the unique challenges faced by this population in managing T2D and improving diabetes-related health outcomes.

The intervention group demonstrated a significant increase in self-efficacy in managing diabetes compared to the control group, and this increase may have reached the threshold for clinical significance. Our findings contribute to the growing body of literature on the effectiveness of mobile health–based interventions aimed at diabetes education and self-management in enhancing self-efficacy, as evidenced in a recent systematic review [38]. Notably, none of the US-based interventions included in the review were specifically tailored for Chinese immigrants, an underserved population with a high burden of T2D and unique barriers to accessing these evidence-based programs [1-4,15-19]. By tailoring the intervention to the cultural and linguistic needs of the Chinese immigrants, the CARE program was able to engage participants in a more meaningful and effective way, which likely increased their confidence in adhering to self-care practices, a key determinant of change in other self-management behaviors [23].

The significant improvement in adherence to a healthy diet in the intervention group compared to the control group further supports the potential of the CARE intervention, particularly in addressing dietary changes, one of the most challenging aspects of T2D management for Chinese immigrants [15]. This improvement may represent a clinically significant change. This group typically follows a traditional Chinese diet [39,40], which is often not reflected in Western dietary guidelines commonly recommended in existing DSMES programs [16]. To address these challenges, the CARE program provided information on culturally appropriate food alternatives for Chinese Americans with T2D. It included video tutorials on reading food labels in local ethnic grocery stores. These strategies could facilitate the adoption of healthier dietary practices in this group. These findings underscore the importance of culturally tailored interventions in diabetes management for this immigrant population.

The intervention group demonstrated a significant improvement in managing diabetes distress compared to the control group. This improvement may be clinically significant. This finding aligns with previous interventional studies, which have reported that diabetes education interventions effectively reduce diabetes distress and offer emotional support to Chinese Americans [31,34]. The improvement in diabetes distress may be attributed to the multifaceted components of the CARE intervention, which integrates diabetes-related knowledge, behavioral techniques, and biweekly phone calls from community health workers to address SDOH concerns. These elements may collectively enhance participants’ self-efficacy in managing diabetes, subsequently alleviating their disease-related distress and other concerns throughout their diabetes journey.

We did not observe significant improvements in other aspects of self-care activities and beliefs in diabetes self-care activities. Several factors may have contributed to these null results. First, the small sample size likely resulted in insufficient statistical power to detect between-group differences in these outcomes. Secondly, the relatively short duration of our intervention (12 weeks) compared to some other DSMES programs (24 weeks) may not have been enough time to produce observable changes in these behavioral aspects of diabetes self-management. A larger-scale RCT with sufficient power is necessary to examine the effectiveness of the intervention across a broader range of diabetes-related outcomes. These findings will also inform the sample size for a future, fully powered RCT to assess CARE’s effectiveness and its potential to generalize these promising results to a broader population.

Several limitations should be noted. First, the sample size for this study was powered for HbA_1c_ levels; therefore, the self-efficacy and self-care activities reported in the current study may not have sufficient statistical power. The small sample size may limit the ability to detect significant differences in some outcomes. However, the primary goal of this pilot RCT was to assess the potential efficacy of the intervention and to generate pilot data for a future large-scale RCT. Second, all outcomes were self-reported and collected by community health workers over the phone and thus are subject to potential recall bias and social desirability bias. Additionally, our findings may not be generalizable to all Chinese Americans, particularly those with different sociodemographic characteristics. For instance, the majority of participants in our study had limited English proficiency, so the results may not apply to more acculturated counterparts who are fluent in English. Despite these limitations, this study is important as it serves as the first step in exploring scalable strategies to disseminate evidence-based diabetes interventions for underserved and minoritized populations. It also has the potential to serve as a care model for delivering culturally and linguistically tailored interventions to other immigrant or minoritized communities facing similar challenges in managing T2D and to lay the groundwork for a larger RCT.

### Conclusions

This study provides promising evidence that culturally and linguistically tailored interventions may improve self-efficacy, dietary adherence, and diabetes distress among Chinese Americans. The use of accessible platforms like social media for health education shows promise in supporting diabetes management. Future research with adequate statistical power is needed to establish the effectiveness of our intervention. By addressing the cultural and linguistic needs of immigrant populations, tailored interventions have the potential to reduce health disparities and improve diabetes-related health outcomes for immigrant or minoritized communities with T2D.

## Appendix

Multimedia Appendix 1 Screenshots of the Chinese American Research and Education (CARE) intervention.

Multimedia Appendix 2 CONSORT 2010 checklist.

## Abbreviations

CARE: Chinese American Research and Education
CONSORT: Consolidated Standards of Reporting Trials
DSMES: Diabetes self-management education and support
HbA_1c_: hemoglobin A_1c_
MCID: minimal clinically important difference
RCT: randomized controlled trial
SDOH: social determinants of health

## Data Availability

The datasets generated or analyzed during this study are available from the corresponding author on reasonable request.
