# Supplementary figures and images for: A Culturally and Linguistically Tailored Intervention to Improve Diabetes-Related Outcomes in Chinese Americans With Type 2 Diabetes: Pilot Randomized Controlled Trial
Source: JMIR Mhealth Uhealth. 2025 Oct 27;13:e78036. doi: 10.2196/78036 (PMC12603588; doi:10.2196/78036)

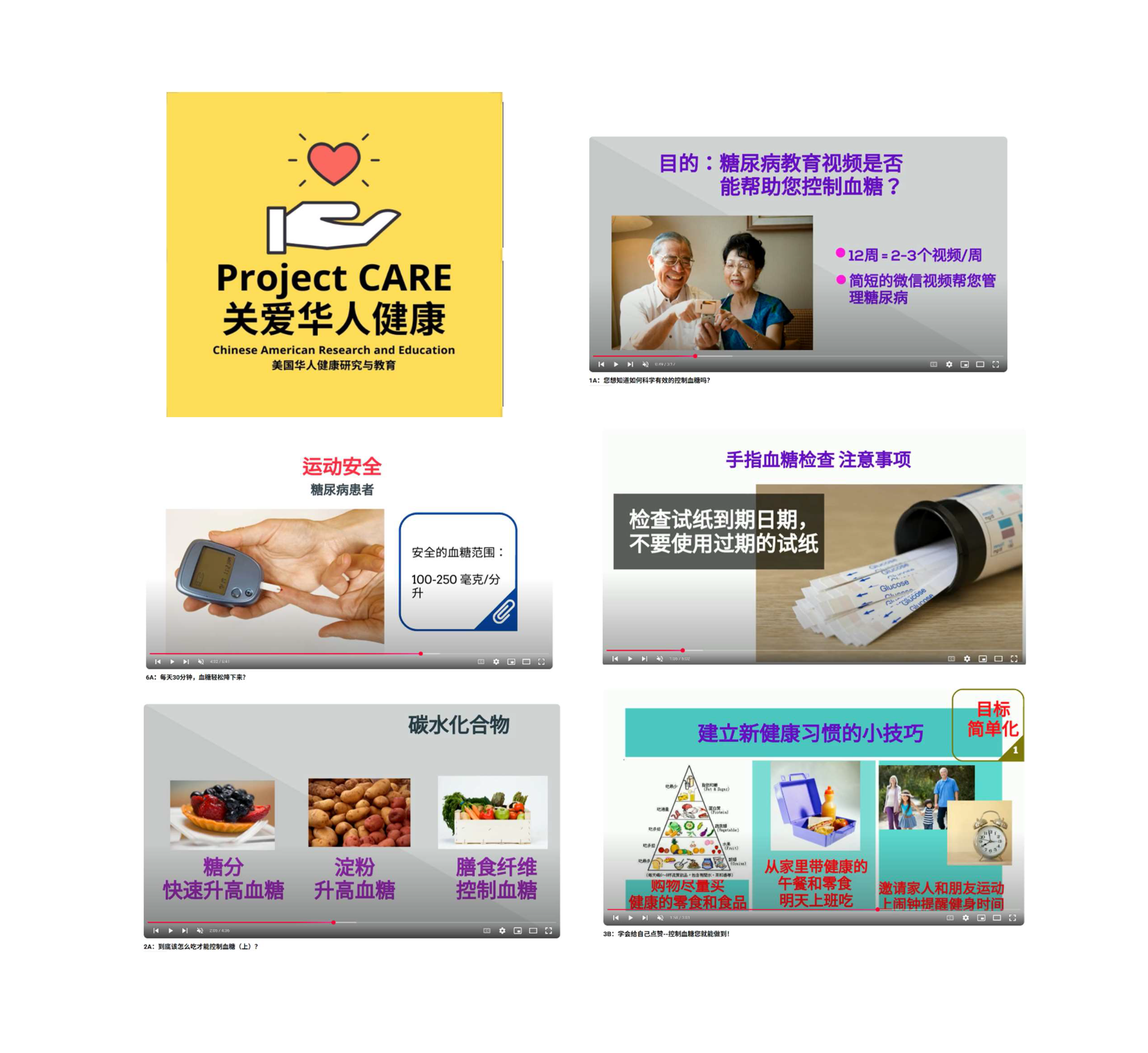

Supplement: Multimedia Appendix 1 [file mhealth_v13i1e78036_app1.png]
